# Printing knowledge in microgravity: PULSE and the education needed for space biomedicine

**DOI:** 10.1113/EP094256

**Published:** 2026-09-29

**Authors:** Sarah Baatout, Michela Candotti, Kevin Tabury, Bjorn Baselet, Damian M. Bailey, Lorenzo Moroni

**Affiliations:** ^1^ Institute of Nuclear Medical Applications SCK CEN (Belgian Nuclear Research Centre) Mol Belgium; ^2^ INSociety Udine Italy; ^3^ Neurovascular Research Laboratory, Faculty of Life Sciences and Education University of South Wales Pontypridd UK; ^4^ Complex Tissue Regeneration department, MERLN Institute for Technology‐Inspired Regenerative Medicine Maastricht University Maastricht the Netherlands

## 1 WHY PHYSIOLOGY MUST TRAVEL WITH TECHNOLOGY

*Humanity can travel only as far as physiology allows*.

Humanity can travel only as far as physiology allows. The history of exploration is often narrated through machines: the vessel, the aircraft, the launch vehicle and, increasingly, the autonomous laboratory. Yet, every technological frontier is also a physiological experiment. When the human body is removed from the terrestrial conditions in which it evolved, familiar systems are asked unfamiliar questions. Microgravity alters mechanical loading, fluid distribution and cellular organisation; radiation can damage DNA and remodel inflammatory and metabolic responses; confinement, disrupted circadian timing and operational stress add further layers of biological and psychological complexity. These influences interact across scales rather than acting as isolated hazards (Afshinnekoo et al., 2020; Bailey et al., 2025).

This is why space biomedicine should not be treated as an accessory to engineering. It is one of the disciplines that determines whether exploration is safe, sustainable and genuinely human‐centred. It also has a reciprocal value for health on Earth. Removing gravity as a dominant physical constraint can expose mechanisms that are difficult to isolate terrestrially, while accelerated or amplified biological responses may help us interrogate ageing, cardiovascular deconditioning, musculoskeletal loss, immune dysregulation and tissue repair. Space is therefore not simply a destination for biomedical research; it is a perturbation that can reveal how deeply physiology depends on context.

The European PULSE project sits at this interface. Its full title – 3D Printing of Ultra‐fideLity tissues using Space for anti‐ageing solutions on Earth – captures a deliberately bidirectional ambition: to use space conditions to advance biofabrication and to return the resulting knowledge to medicine on Earth. Funded through the European Innovation Council Pathfinder Open programme under grant agreement 101099346, PULSE runs from April 2023 to March 2028 and brings together expertise in biofabrication, tissue engineering, cardiology, radiation biology, pharmacology and space operations (European Commission, 2026). Its educational programme deserves attention because the scientific problem it addresses cannot be solved by any one of these fields alone. This science‐to‐community pathway – linking space conditions, advanced biofabrication, physiologically relevant 3D tissue models and discoveries for health on Earth – is summarised in Figure 1.

**FIGURE 1:**
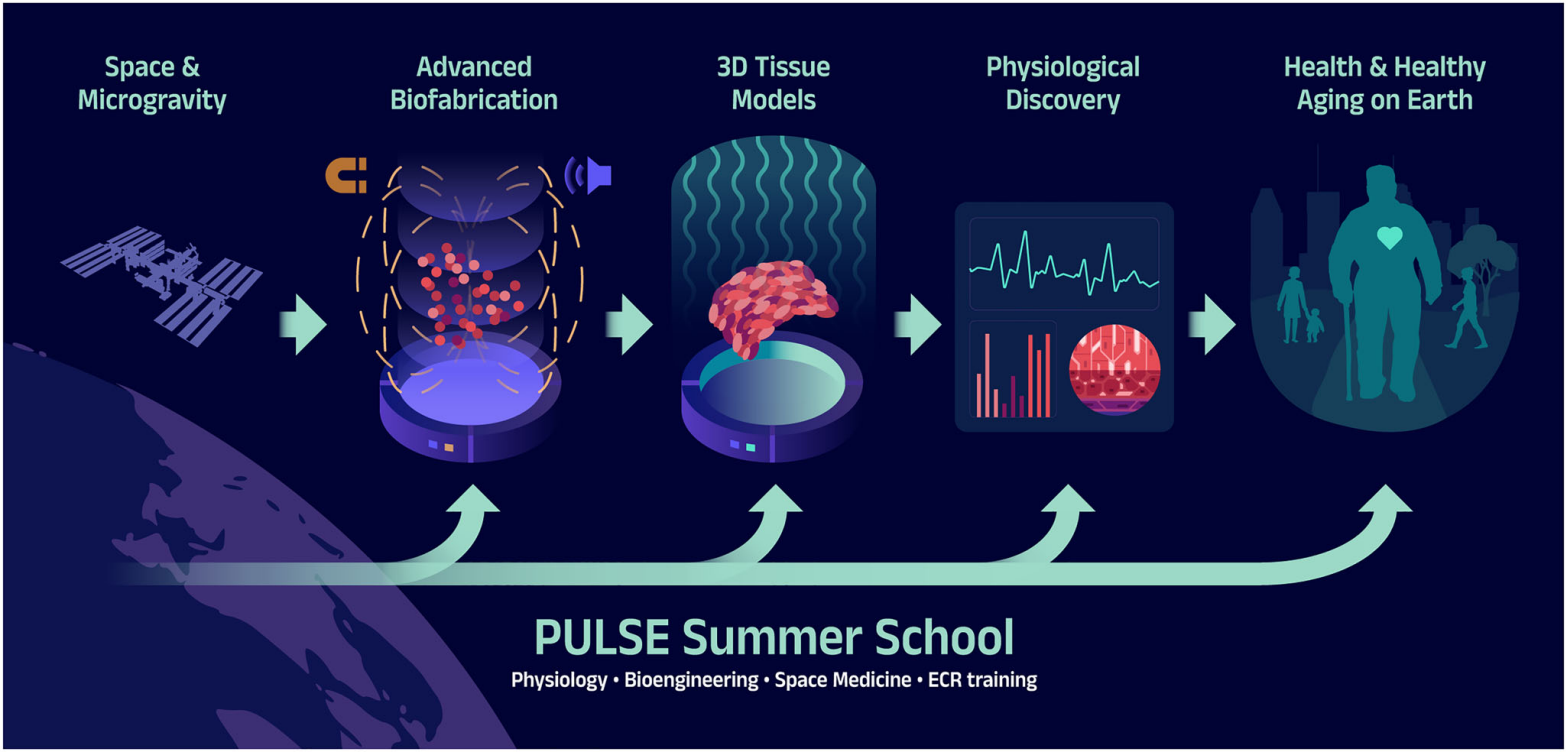
The PULSE science‐to‐community pathway. Space and microgravity provide new conditions for advanced biofabrication and the generation of physiologically relevant 3D tissue models. These models enable discoveries that can inform human health and healthy ageing on Earth. The PULSE Summer School supports this pathway by connecting physiology, bioengineering and space medicine while equipping early‐career researchers to contribute to the emerging field.

## 2 PULSE: A PHYSIOLOGICAL QUESTION EMBEDDED IN A BIOENGINEERING PLATFORM

At the technological core of PULSE is a magneto‐acoustic approach to bioassembly. Magnetic and acoustic levitation are being combined to position and manipulate cells and biomaterials with high spatiotemporal control. The objective is a scaffold‐free, label‐free and nozzle‐free platform capable of generating complex multi‐tissue constructs. Microgravity is scientifically attractive in this context because reduced sedimentation and altered buoyancy may permit the assembly of geometries and fluidic architectures that are difficult to achieve on Earth. The promise is substantial, but it should be expressed cautiously: microgravity does not remove the fundamental challenges of tissue maturation, vascularisation, reproducibility, functional validation or translation. It changes the physical boundary conditions under which those challenges can be studied (European Commission, 2026; Tabury et al., 2023).

PULSE uses three‐dimensional human cardiac models as an initial proof of concept. The choice is physiologically compelling. The cardiovascular system is highly responsive to altered loading, fluid shifts, autonomic regulation and radiation exposure (Baselet et al., 2024; Rehnberg et al., 2024). A model that better recapitulates cardiac architecture and function could help disentangle how spaceflight‐relevant stressors influence cellular communication, electrophysiology, contractility, metabolism and inflammatory signalling. It could also provide a platform for testing candidate protective or therapeutic interventions. Importantly, such models complement rather than replace human studies, animal models or established ground‐based analogues. Their value will depend on rigorous benchmarking, transparent reporting and evidence that increasing structural complexity produces greater physiological relevance.

This balance between technological ambition and biological validation is precisely where integrative physiology contributes. A construct may look anatomically persuasive, yet fail to reproduce a meaningful systems‐level response. Conversely, a relatively simplified model may answer a carefully defined mechanistic question extremely well. Physiologists help frame those questions: which function is being modelled, which response is expected, which comparator is appropriate and what degree of concordance is sufficient for a stated use? PULSE therefore illustrates a broader lesson for emerging biomedical technologies. Innovation is not completed when a platform can be built; it becomes useful when its outputs can be interpreted within the living system. For *Experimental Physiology*, this is the critical connection: technological sophistication has value only when structural fidelity can be linked to measurable physiological function.

## 3 THE SUMMER SCHOOL AS AN INTERDISCIPLINARY LABORATORY

The PULSE Online Summer School, held from 25 to 29 August 2025, translated this scientific architecture into an accessible educational programme. More than 500 students, early‐career researchers and professionals participated in a free 5‐day event devoted to tissue engineering and space medicine. The programme moved deliberately across levels of organisation and disciplinary languages: muscle, bone, immunity, reproduction, the cardiovascular system and the brain were considered alongside microfluidics, preservation, hydrogels, microgravity simulation, extrusion bioprinting, magneto‐acoustic levitation, radiation protection and European Space Agency career pathways (PULSE, 2025).

The sequence matters. A lecture on radiation resistance acquires a different meaning when placed beside sessions on cardiac model preservation and highly complex radiation fields. A discussion of lymphopoiesis or reproductive biology changes when connected to biofabrication and mission constraints. Engineering students encounter the biological consequences of design choices; physiologists see how experimental questions are constrained by payload mass, automation, containment and access to microgravity; clinicians are invited to consider how an extreme environment can sharpen questions relevant to patients on Earth. The educational value lies not simply in accumulating topics but in learning to connect them.

The online format also lowered geographical and financial barriers. Recorded lectures extend the life of a time‐limited school and allow learners to revisit unfamiliar concepts at their own pace. The 2026 PULSE Space Summer Week, held on 24–27 August, continues this model through free lectures on space biology, space medicine, bioprinting and immune responses in microgravity (PULSE, 2026). The online platform, including the 2026 programme and repository of recordings from the 2025 Summer School, is illustrated in Figure 2. Such openness is important in a field where access to specialist courses, laboratories and spaceflight platforms remains uneven.

**FIGURE 2:**
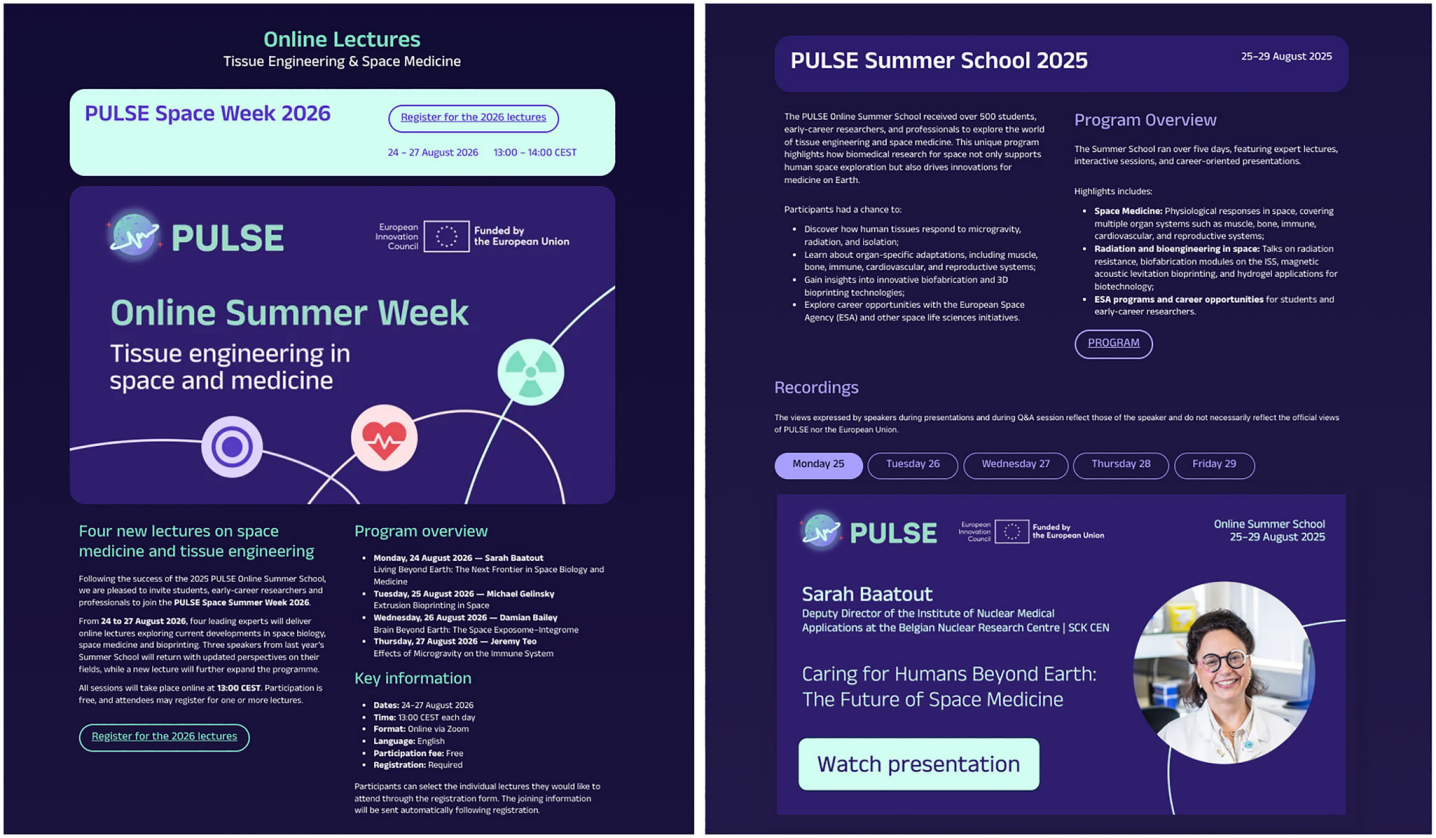
The PULSE online lecture and recording platform. Screenshots from the PULSE Online Lectures webpage, showing the 2026 Space Week programme and the repository of recordings from previous editions of the PULSE Summer School. The freely accessible lectures provide a lasting educational resource for students, early‐career researchers and professionals interested in tissue engineering and space medicine. Recordings are available at: https://pulse‐eic.eu/online‐lectures/ (accessed 26 August 2026).

Accessibility, however, should not be confused with complete training. Online lectures cannot by themselves confer experimental competence, teach the tacit judgement required at the laboratory bench or reproduce the creative friction of sustained interdisciplinary teamwork. They are best understood as an entry point and a connective layer: a means of building shared vocabulary, exposing learners to possible career trajectories and helping them identify the questions for which deeper mentorship or hands‐on experience is needed. Future iterations could strengthen this pathway through case‐based group work, structured mentoring, small interdisciplinary design challenges, and transparent evaluation of learning and longer‐term career impact. More broadly, PULSE may provide a scalable model for space‐biomedicine training, in which no single discipline can equip researchers for the scientific, technological and translational complexity of the field.

## 4 EDUCATING FOR CURIOSITY, RESPONSIBILITY AND TEAM SCIENCE

Education at a scientific frontier must do more than transfer established knowledge. It must cultivate the capacity to ask good questions when evidence is incomplete. Space research makes this need unusually visible: sample sizes are often small, access is scarce, biological variability is considerable and operational constraints can determine what is measurable. Early‐career researchers must therefore learn to distinguish possibility from proof, technological readiness from physiological validity and an attractive narrative from a testable hypothesis.

This is also an education in responsibility. Human tissues, radiation exposures and spaceflight experiments each bring ethical, safety and governance obligations. The attraction of exploration should not eclipse the need for reproducibility, appropriate controls, careful uncertainty language and responsible stewardship of public funding. Nor should the promise of translation to Earth be assumed. It must be demonstrated through comparisons that establish when and for whom a space‐enabled model adds value.

Interdisciplinarity is often invoked as an aspiration, but productive team science requires explicit skills: listening across vocabularies, making assumptions visible, negotiating standards of evidence, sharing credit and recognizing the limits of one's own methods. These are not secondary professional attributes. They shape whether complex collaborations succeed. Early‐career researchers (ECRs) can bring intellectual flexibility and fresh combinations of expertise, yet they also need mentoring, recognition and clear routes to authorship and career progression in team‐based work (National Research Council, 2015). Importantly, ECRs should be viewed not simply as recipients of training, but as contributors who can help define the questions, methods and collaborative culture of this emerging field.

The deepest educational contribution of PULSE may therefore be cultural. It presents the body not as a collection of independent parts, nor technology as an external solution imposed upon biology, but rather both as components of a coupled system. This view echoes a central lesson from space medicine: health is not static. It is a dynamic equilibrium produced through continuing interaction among the body, its environment and the tools humans create to inhabit that environment. By taking physiology outside its familiar context, we see more clearly what sustains resilience – and what can disrupt it.

## 5 FROM A COURSE TO A COMMUNITY

PULSE should be judged ultimately by the quality and reproducibility of its science, not only by the novelty of its setting. Its educational activities should likewise be assessed not only by attendance but also by whether learners acquire durable understanding, form productive new collaborations and gain equitable access to further opportunities. Participation metrics, disciplinary and geographical reach, feedback, follow‐up training and subsequent research engagement would help establish what works and where the model should evolve.

Nevertheless, the first Summer School demonstrates what an EU Pathfinder project can contribute beyond its technical deliverables. It can make a developing field legible to newcomers, connect communities that rarely interact and invite young researchers to participate before disciplinary boundaries become fixed. That is not promotional activity at the edge of science; it is part of building the human capital and capability on which future science depends. Success would ultimately be reflected not simply in attendance, but in a community able to progress from accessible interdisciplinary training to sustained mentorship, hands‐on research and collaborative space‐biomedicine projects. More broadly, the value of PULSE should be measured in both directions: by what space reveals about human physiology and by how effectively that knowledge is translated back to improve health on Earth.

We therefore invite physiologists to engage actively with biofabrication and space research; educators to make interdisciplinary training accessible, critical and evaluable; and students and early‐career researchers to bring their questions, ideas, methods and healthy scepticism to this emerging community. The next generation should not be trained merely to operate the technologies of exploration. It should be equipped to decide which technologies are physiologically meaningful, ethically responsible and worthy of carrying human health beyond Earth – and bringing knowledge home.

## AUTHOR CONTRIBUTIONS

Sarah Baatout conceived the editorial and prepared the initial manuscript. Lorenzo Moroni coordinated the project and provided biofabrication expertise. Michela Candotti, Kevin Tabury, Bjorn Baselet and Sarah Baatout organised the 2025 and 2026 PULSE Summer Schools and contributed their insights and experience to the editorial. Damian M. Bailey contributed to the editorial conception and physiological interpretation. All authors critically revised the manuscript. The authors affirm that they have read and approved the final version of the manuscript and agree to be accountable for all aspects of the work, ensuring that any questions related to the accuracy or integrity of any part of the work are appropriately investigated and resolved. All individuals designated as authors meet the criteria for authorship, and all individuals who meet these criteria are listed as authors.

## CONFLICT OF INTEREST

Damian M. Bailey is Editor‐in‐Chief of *Experimental Physiology*. The other authors declare no conflicts of interest.

## GENERATIVE AI STATEMENT

OpenAI ChatGPT was used for limited language editing and for preparing the initial concept for Figure 1, which was then subsequently reviewed, adapted and finalised by INsociety based on the scientific content and visual identity of PULSE.
